# Predictors of Retirement Voluntariness Using Canadian Longitudinal Study on Aging Data

**DOI:** 10.1177/08982643241229760

**Published:** 2024-01-30

**Authors:** Mary Beth MacLean, Christina Wolfson, Sarah Hewko, Emile Tompa, Jill Sweet, David Pedlar

**Affiliations:** 1 12363 Queen’s University, Kingston, ON, Canada; 2 5620 McGill University, Montreal, QC, Canada; 3 2359 University of Prince Edward Island, Charlottetown, PE, Canada; 4 7938 University of Toronto, Toronto, ON, Canada; 5 26621 Veterans Affairs Canada, Charlottetown, PE, Canada; 6 Scientific Director of the Institute for Military and Veteran Health Research, 4257 Queen’s University, Kingston, ON, Canada

**Keywords:** Canadian Longitudinal Study on Aging, life course perspective, retirement voluntariness, predictors, involuntary retirement

## Abstract

**Objectives:** Involuntary exit from the labor force can lead to poor health and well-being outcomes. Therefore, the purpose of this research is to better understand the factors that contribute to perceived retirement voluntariness. **Methods:** We conducted descriptive and multivariable logistic regression analyses using a sample of recent retirees (*n* = 2080) from the Canadian Longitudinal Study on Aging (CLSA). **Results:** More than one-quarter (28%) of older workers perceived their retirement to be involuntary. Among 37 possible predictors, 14 directly predicted retirement voluntariness and many more indirectly predicted retirement voluntariness. Only four direct predictors were common to both women and men, retiring because of organizational restructuring/job elimination; disability, health, or stress; financial possibility; and having wanted to stop working. **Discussion:** Findings suggest the need for employment support, health promotion, work disability prevention, financial education, and support that is sensitive to the differences between women and men to prevent involuntary retirement.

## Introduction

The transition into retirement is an important life course event. Most Canadians retire voluntarily and adjust well. However, it is estimated that between 20 and 30% of Canadians retire involuntarily (Schellenberg & Silver, 2004; Schirle & Veall, 2018). Those who perceive their retirement as involuntary generally experience poor outcomes such as reductions in life satisfaction in older age, difficulties in adjusting to retirement (Dingemans & Henkens, 2014, 2015; Hershey & Henkens, 2014), depression (Li et al., 2021; Rhee et al., 2016), and adoption of unhealthy lifestyles, including problematic drinking (Bacharach et al., 2008). For the wider society, implications of retirement in general and involuntary retirement may include labor shortages in some industries/occupations, risks to the sustainability of public and private pension plans, and reduced tax revenue (OECD, 2015; Rouzet et al., 2019).

In Canada, older workers account for an increasingly large part of the labor force, with more than one-third of aged 55 and older in 2016; by 2026, that proportion is expected to rise to 40% (Fields, 2017). A significant proportion of these workers may be at risk of being forced out of the labor force due to job loss, their own health issues or disability, and/or caregiving responsibilities for a spouse or aging parents (Denton et al., 2013; Humble et al., 2012). Thus, there are implications for employers, the health care system, and policy makers concerned with the sustainability of public finances and pensions and the labor market in general.

While many older Canadians are willing to remain in the labor market and there are interventions to extend working lives (Carrière & Galarneau, 2011; Phillipson, 2019; Wainwright et al., 2019), our research found important gaps in our knowledge regarding who is at risk of involuntary retirement (MacLean et al., 2022). While several theoretical models have been developed on predictors of involuntary retirement (Denton et al., 2013; Hewko, 2018; Shultz et al., 1998; Szinovacz & Davey, 2005) and each model has its own strengths and weaknesses, in general they are missing key factors that pull individuals into retirement such as having hobbies and retirement being financially possible. Further, these factors are not structured around the principles of the life course perspective (Elder, 1995; Elder et al., 2003)—that is, human agency, life-span development, linked lives, timing, and time and place—essential to understanding retirement decision-making (Mortimer & Shanahan, 2003). Moreover, there are a limited number of studies on factors associated with perceived retirement voluntariness, that is, the retiree’s perception of the degree to which he or she retired voluntarily (Beehr, 1986). In fact, a recent systematic literature review found only nine studies across five databases (MacLean et al., 2022). Perhaps most importantly, previous studies have not performed sex-disaggregated analyses (MacLean et al., 2022), even though women and men differ in their labor market participation. For example, women are more likely to have breaks in labor-market participation due to caregiving responsibilities (Berger & Denton, 2004; Martinengo et al., 2010; Moyser, 2017), to receive lower wages, and to work part-time (Moyser, 2017). For these reasons, their retirement decisions are likely to differ from those of men. Finally, despite the significant proportion of Canadians perceiving their retirement as involuntary and the nature of retirement as a life course process, there have been no nationally representative longitudinal studies of retirement in Canada.

The objectives of this research are to develop a more comprehensive theoretical model of retirement voluntariness and to test hypotheses based on this model using nationally representative longitudinal Canadian data of recent retirees and sex-disaggregated analysis. The goal is to inform involuntary retirement prevention and mitigation efforts, ultimately improving retirement outcomes while also leading to more sustainable public finances and the alleviation of labor shortages in some industries and occupations.

## Theoretical Model

### Concept of Retirement Voluntariness

We adopted the concept of “retirement voluntariness” proposed by psychologist Terry Beehr (1986). Beehr conceptualized it as “the retiree’s perception of the degree to which he or she retired voluntarily” (Beehr, 1986, p. 34). This conceptualization of voluntariness as a matter of perception has been used in several studies (Shultz et al., 1998; Swan et al., 1991; Szinovacz & Davey, 2005). The Canadian Longitudinal Study on Aging (CLSA) also used a measure of voluntariness based on perception. Respondents who report they were completely or partly retired were asked “*Would you say your retirement was voluntary, that is, you retired when you wanted to?*” with response categories of “*yes*” or “*no.*”

The life course perspective is particularly relevant to exploring the factors explaining retirement voluntariness as it provides for a framework to categorize predictors into various life domains impacting the retirement decision (Elder, 1995; Elder et al., 2003). The first of five principles of the life course perspective, human agency, refers to individuals constructing their own life course through the choices they make and actions they take within the opportunities and constraints of history and social circumstances (Mortimer & Shanahan, 2003). Therefore, in this study, human agency represents voluntariness in the decision to retire. The remaining four principles act as opportunities and constraints in the decision to retire: life-span development (human development—biological, psychological, and sociological—and aging are lifelong processes); linked lives (lives that are lived interdependently, and socio-historical influences are expressed through this network of shared relationship); timing (the developmental antecedents and consequences of life transitions, events, and behavioral patterns vary according to their timing in a person’s life); and time and place (the life course of individuals is embedded and shaped by the historical times and places they experience over their lifetime) (Mortimer & Shanahan, 2003).

### Models and Factors Explaining Retirement Voluntariness

We found three theoretical models of retirement voluntariness in the peer-reviewed literature. All three focused on the outcome of perceived involuntary retirement rather than the broader concept of retirement voluntariness. With a focus on involuntary retirement, choice factors are limited to those that restrict the retirement decision, often thought of as push factors. Therefore, these theoretical models were missing pull factors (e.g., desire to pursue leisure interests) identified in previous studies of retirement voluntariness (De Preter et al., 2013; Hofäcker et al., 2016; Shultz et al., 1998). Furthermore, each model also had its own strengths and weaknesses. First, Szinovacz and Davey (2005) developed the Theoretical Model of Predictors of Forced Retirement Perceptions. While their model included the most comprehensive set of factors explaining perceived forced retirement, it depicted health and job loss as “no choice” factors. While there are certainly many barriers faced by older workers, with proper supports, health and job loss do not necessarily need to lead to retirement. Denton and Colleagues (2013) proposed the Conceptual Model of Predictors of Perceptions of Involuntary Retirement. This model, based on the Szinovacz and Davey model, focused on the retirement decisions of people with disabilities. Unlike the Szinovacz and Davey model, theirs distinguished between the effects of health and disability in the decision to retire. However, work context factors were not included in their model. Finally, Hewko and colleagues (2018) proposed the Model of Involuntary Retirement among Nurses and Allied Health Professionals (Hewko et al., 2018). While this model distinguished between mental and physical health as well as health and disability, human capital and finances factors were missing. Further, this theoretical model was built for the study of involuntary retirement among a subset of workers in Canada, that is, nurses and allied health professionals.

### Proposed Theoretical Model

**Figure 1.**
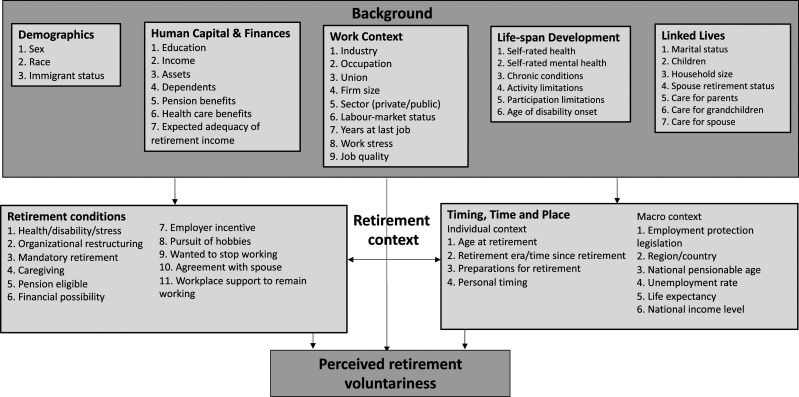
Theoretical model of predictors of perceived retirement voluntariness.

We started with the Szinovacz and Davey model as it included the most comprehensive set of predictors. We changed the outcome of interest to perception of retirement voluntariness instead of involuntary retirement to allow for factors often considered to be associated with voluntary retirement to be included and as this change was consistent with our conceptualization of voluntariness as a matter of degree (Figure 1). To better align our model with life course perspective principles, we added two domains under background factors—that is, life-span development and linked lives—and renamed two domains, that is, from restricted choice to retirement conditions (i.e., factors that restrict or provide more latitude for choice in the decision to retire) and from retirement context to timing, time, and place. In our model, background factors reflect circumstances that would have been in place prior to the retirement decision. These factors can directly or indirectly influence the perception of retirement voluntariness. Retirement context factors, which include the domains of “retirement conditions” and “timing, time, and place,” are related to the retirement decision itself and therefore have a direct influence on the perception of retirement voluntariness. Further, factors within these domains interact with each other in the decision to retire. For example, having to leave work due to health or disability may interact with age at retirement such that if the timing of retirement is “on-time” and the odds of the perception of involuntary retirement may be reduced. Next, we added factors to explain retirement voluntariness, as identified in a recent systematic literature review (MacLean et al., 2022). In total, our model includes 53 predictors of perceived retirement voluntariness categorized into seven domains, compared to the Szinovacz and Davey model which depicted 20.

#### Hypotheses

In constructing our hypotheses, we needed to consider that while our adopted definition and model was based on predictors of the degree of perceived retirement voluntariness, previous research and the data on which we were testing our model used a dichotomous measure of voluntariness. Therefore, our hypotheses have been constructed to match this dichotomous conceptualization of voluntariness.

We considered several hypotheses related to background and retirement context factors. First, since women and men differ in their labor market experiences (Berger, 2021; Lightman & Gingrich, 2012) and are subject to gendered roles, we expect predictors of retirement voluntariness to differ by sex. Second, as minority groups and those who immigrated to Canada experience greater economic exclusion in the labor market (Lightman & Gingrich, 2012), it is not surprising that previous studies have found blacks (Szinovacz & Davey, 2005) and immigrants (Denton et al., 2013; Ebbinghaus & Radl, 2015) experience greater odds of involuntary retirement. Therefore, we expect that those who are non-white or immigrated to Canada will be more likely to perceive their retirement as involuntary. Third, in line with previous studies, we expect that lower education (Denton et al., 2013; Humble et al., 2012; Szinovacz & Davey, 2005) and poorer finances will lead to greater odds of involuntary retirement (Humble et al., 2012; Shultz et al., 1998; Szinovacz & Davey, 2005). Fourth, work comes with both rewards and costs. In this regard, greater odds of perceived involuntary retirement have been found among those who worked in the private sector, mining, as well as in sales and service (Ebbinghaus & Radl, 2015; Szinovacz & Davey, 2005), non-managerial and non-professional (Shultz et al., 1998) occupations. Members of these occupations could potentially have a greater risk of work-related hazards and/or likelihood of job loss. Moreover, after job loss, older workers experience greater difficulty searching for new employment as compared to younger workers (Berger, 2012, 2021). Therefore, we expect that those who have lost their jobs or report being not able to work will experience barriers to re-entering the labor market, leading them to be more likely to perceive their retirement as involuntary. Fifth, over the life span many people acquire chronic conditions that lead to disability and poor health. It has been well established that these factors are linked to involuntary retirement (MacLean et al., 2022). Thus, we expect that disability or poor health will lead to greater odds of perceived involuntary retirement. Finally, the decision to retire is not an individual decision but one that is interdependent with others. Therefore, as has been found by Szinovacz and Davey (2005), we expect that having a spouse who is not retired, as couples often retire together, and having caregiving responsibilities will lead to greater odds of perceived involuntary retirement.

Besides background factors, the context surrounding retirement has been found to predict retirement voluntariness. First, conditions linked to greater odds of the perception of involuntary retirement include disability and health (Denton & Spencer, 2009; Ebbinghaus & Radl, 2015; Humble et al., 2012; Szinovacz & Davey, 2005), job loss (Ebbinghaus & Radl, 2015; Humble et al., 2012; Szinovacz & Davey, 2005), and caregiving responsibilities (Humble et al., 2012; Szinovacz & Davey, 2005; Welsh et al., 2018). We expect these same conditions surrounding retirement to result in greater odds of involuntary retirement. Second, we expect that retiring due to being eligible for a pension, as it was financially possible, and to pursue hobbies and other activities will result in greater odds of voluntary retirement. Third, in terms of timing, time, and place of retirement, as found in previous studies, we expect that workers who retired at younger ages (<65) (Denton & Spencer, 2009; Szinovacz & Davey, 2005), or retired more recently and therefore had less time to adjust to retirement, will have greater odds of involuntary retirement (Dorn & Sousa-Poza, 2010; Hofacker et al., 2016; Steiber & Kohli, 2017). On the other hand, we expect that those who reported having prepared for retirement will have greater odds of voluntary retirement (Shultz et al., 1998). Finally, as the unemployment rate has typically been higher in Atlantic Canada, we expect that retirees in that region will be more likely to experience involuntary retirement.

## Methods

### Data Source

In Canada, the Canadian Longitudinal Study on Aging (CLSA) is the best data source for studies on retirement as it includes a large sample of workers that can be followed as they move through the retirement process. The CLSA is a national, longitudinal research platform that includes participants from all 10 Canadian provinces and collects comprehensive data and biological samples (Raina et al., 2009). The cohort of 51,338 participants, aged 45–85 years at enrollment, is composed of the Tracking Cohort of 21,241 participants and the Comprehensive Cohort of 30,097 participants. In addition, the sampling and sampling weights ensure rural, urban, age-sex, and national representation. All participants are being followed up every three years after baseline until 2033 or until death.

In this study, we have used baseline (version 3.7, *n* = 21,241) and follow-up one (version 2.2, *n* = 17,052) data collected as part of the Tracking Cohort questionnaire (https://clsa-elcv.ca/doc/446) as members of the Comprehensive Cohort are not asked about retirement voluntariness. The questionnaire is administered by telephone and includes detailed questions on the conditions surrounding retirement (retirement status, reasons for retirement, work context, and retirement planning). While some groups were excluded at baseline, such as institutionalized individuals and those with cognitive impairments, these exclusions represent a small proportion of the Canadian population. Further details are available at https://clsa-elcv.ca/doc/511.

### Study Sample

As older workers contemplating retirement today face a very different environment than prior cohorts did (Cahill et al., 2015) and to take advantage of available longitudinal data with potential predictors of retirement voluntariness, our study sample consisted of individuals who had more recently retired, that is, those who reported they were not retired at baseline (2011–15) and reported they were retired at follow-up one (2015–18). This sample consists mainly of baby boomers (84%)—that is, individuals born between 1946 and 1965 (Statistics Canada, 2012)—who would have been between the age of 46 and 65 in 2011.

Retirees were identified with the following question at baseline and follow-up one: “*At this time, do you consider yourself to be completely retired, partly retired, or not retired?*” In terms of voluntariness, retirees were asked the following question: “*Would you say your retirement was voluntary, that is, you retired when you wanted to?*” The answer categories were yes or voluntary (=0) and no or involuntary (=1).

**Figure 2.**
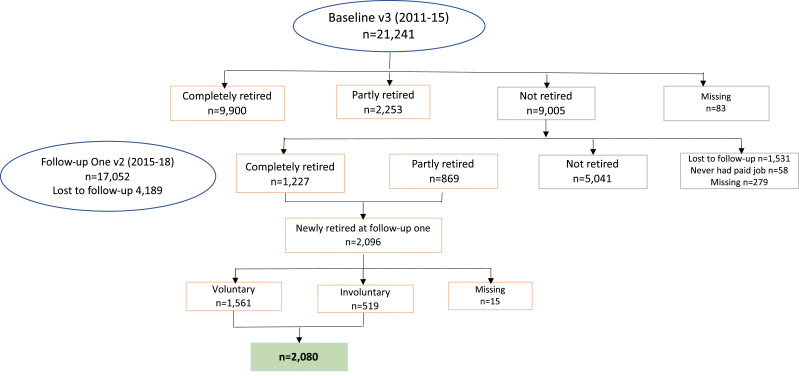
Study sample.

Among the 21,241 respondents to the CLSA Tracking Cohort at baseline (2011–15), 83 did not respond to the question on retirement status and were therefore removed from our sample (Figure 2). Of the remaining 21,158 participants, 9005 had not retired. From the sample who had not retired at baseline, we included those who reported they either completely or partly retired at follow-up one (*n* = 2096). After removing those with missing data on retirement voluntariness, our final sample had 2080 individuals, of whom 519 reported that they retired involuntarily.

### Measures

Our outcome variable of interest was retirement voluntariness as described above. Among the 53 predictors of retirement voluntariness in our theoretical model, 37 were available in the CLSA. The following provides a description of each variable categorized into the seven domains of our model: demographics, human capital and finances, work context, life-span development, and linked lives (background factors) and retirement conditions and timing, time, and place factors (retirement context).

#### Demographics

Our model includes three demographic factors that explain retirement voluntariness, all of which were available in the CLSA. We included baseline demographic data for sex (male and female), race (white and non-white), and immigration status (immigrant and non-immigrant), with female, white and non-immigrant as the reference groups.

#### Human Capital and Finances

Four of the seven human capital and finances factors in our model were captured in the CLSA: education, household income, pension benefits, and expected adequacy of retirement income. Data on assets or wealth, dependents, and health care benefits were unavailable. Education (assessed at baseline) was categorized into highest level as less than high school, high school graduate, some post-secondary, and post-secondary graduate, with less than high school as the reference category. Three variables represented finances in our analysis: household income, having employer pension benefits, and expected adequacy of income in retirement. Pre-retirement household income was categorized as <$50,000, $50,000 to <$100,000, $100,000 to <$150,000, and $150,000 or more, with the lowest income as the reference group. For the models, the three non-reference categories were converted to three dummy variables of comparison to the reference category. Pension benefits, from baseline data, were categorized into employer pension benefits and no employer pension benefits. Expected adequacy of retirement income at baseline was categorized as adequate, barely adequate, and inadequate, with adequate serving as the reference category.

#### Work Context

Our model set out eight work context factors, of which four were available in the CLSA. We included baseline data for employment status, years working at last job, occupation, and industry. Employment status was categorized as employed, unable to work because of sickness or disability, unemployed, and involved in non-work activities such as looking after family, education, and volunteer work, with employed as the reference category. Years at last job was categorized as follows: <1 year, 1 to <3 years, 3 to <5 years, and 5 years or more, with 5 years or more as the reference. Industry and occupation were in free text format in the CLSA and were coded using the North American Industry Classification System (Statistics Canada, 2018) and the National Occupation Classification (Statistics Canada, 2016). Education/law and government was used as the reference category for occupation and public administration for industry.

#### Life-Span Development

Five of the six life-span development factors in our model were available in the CLSA. Due to small numbers, self-rated health and self-rated mental health at baseline were coded into three categories: excellent/very good, good, and fair/poor with excellent/very good as the reference. More than 35 chronic conditions were included in the CLSA. We coded chronic conditions for descriptive analysis into one condition, two conditions, three to four conditions, and five plus conditions and for modeling as a continuous variable of number of chronic conditions. The number of chronic conditions has been found to be associated with hospitalizations (Wang et al., 2020) and therefore provides for an indicator of severity of illness. Our chosen measures of disability were consistent with the social model of disability, that is, they recognize the role of environmental factors in the relationship between body function and structure, daily activities, and social participation. Disability was represented by two variables: activity limitations (limitation in an activity of daily living (ADL) or an instrumental activity of daily living (IADL)) (Fillenbaum, 2016; Fillenbaum & Smyer, 1981) and participation limitations. Activity limitation was measured using a CLSA-derived variable which categorizes the respondent’s ability to perform ADLs and IADLs based on the number of times they indicated that they needed help with an activity or were completely unable to do an activity. The classification values ranged from zero (no problems performing activities of daily living) to four (complete inability in performing daily activities). Given small numbers, it was necessary to dichotomize this variable into no limitations and mild, moderate, or severe limitations with the reference as mild, moderate, or severe limitations. Participation limitations were categorized into no desire to participate in more activities, desire but limited by health, and desire but limited by other reasons with no desire to participate in more activities as the reference category. The sixth factor in our model—age at disability onset—was not available in the CLSA.

#### Linked Lives

All seven of the linked lives factors in our model were available in the CLSA. Marital status, children, household size, and spouse employment status were coded as dummy variables with two categories coded as one and zero, respectively: married/common law and single/separated/widowed, those who have any children versus those who do not, three or more in the household and less than three, and spouse retired and spouse not retired. The CLSA includes a question on the provision of care assistance due to health over the past 12 months followed by a question regarding who received care. These two questions were combined to create four variables: provided assistance, assistance to spouse/partner, assistance to parent, and assistance to others, with did not provide assistance or only provided financial assistance as the reference category for each variable.

#### Retirement Conditions

Our model included 11 conditions that push and pull individuals into retirement, 10 of which were available in the CLSA. CLSA participants were asked about their reasons for retirement through the following question: “Which of the following reasons contributed to your decision to retire?” Response categories were as follows: “completed the required years of service to qualify for pension,” “retirement was financially possible,” “disability, health, or stress reasons,” “employer offered special incentives to retirement,” “organizational restructuring or job eliminated,” “providing care to a family member or friend,” “employer had a mandatory retirement policy,” “wished to pursue hobbies or other activities of personal interest,” “wanted to stop working,” and “an agreement with your spouse or partner.” Respondents could select as many categories as applied to them. Separate variables were created for each of these reasons and coded as one for reason selected and zero for reason not selected.

#### Timing, Time, and Place

Our model included four individual level and six macro factors related to timing, time, and place, of which three individual level factors (i.e., age at retirement, time since retirement, and preparations for retirement) and one macro level factor (i.e., region of residence) were available in the CLSA. Respondents’ age at retirement, captured at follow-up one, was categorized as <55, 55 to 64, and 65 and older, with 65 and older as the reference. Time since retirement was calculated as the difference between age at follow-up one and reported age at retirement and categorized into < one year, one to two years, and three years or more years, with three or more years as the reference. A derived variable, captured from baseline data, was created for preparations for retirement; it categorizes various types of preparations for retirement into two categories: at least some preparations (coded as one) and no preparations (coded as zero). Region was categorized as Atlantic, Quebec, Ontario, Prairies, and British Columbia, with Ontario as the reference category.

### Analysis

We performed both descriptive and logistic regression analysis in relation to predictors of retirement voluntariness among older workers. The descriptive analysis was presented in a table of characteristics of the study sample by sex. This table included weighted valid proportions (i.e., excludes missing data) using inflation weights (CLSA, 2020) and 95% confidence intervals. Inflation weights account for probability of selection into the sample and survey non-response and are used for parameter estimation (CLSA, 2020). As samples are stratified by province, we used inflation weights to maintain national representation of our estimates.

Multivariable logistic regression analysis was chosen because the outcome of interest—voluntariness of retirement—was dichotomous. We used analytic weights in modeling to maintain national representation of our estimates. These weights account for probability of selection into the sample and survey non-response and are for use in examining relationships between variables (CLSA, 2020). Three sets of models were produced: that is, Model 1: unadjusted; Model 2: adjusted, multivariable, controlling for variables within seven groups of predictors (demographics, human resources and finances, work context, life-span development, linked lives, retirement conditions, and timing, time, and place); and Model 3: fully adjusted, controlling for significant variables from Model 2. All models were produced separately by sex. We used a backward selection process to eliminate variables with a *p*-value of more than 0.05. Model 1 produced a large count of statistically significant variables but does not consider the possibility of spurious associations. This was less problematic for final Model 3.

The number of years working at last job and spousal retirement status were dropped between Model 1 and Model 2, as they were highly correlated with employment status and marital status, respectively. We retained marital status as it covered the entire sample, whereas spousal employment status only covered those married. In addition, we retained employment status as being unable to work and unemployment have been more commonly found to predict involuntary retirement than years at last job. Results are shown as odds ratios with confidence intervals at 95% and predictors with *p*-values of less than 0.05 were considered significant.

The analysis also included a collinearity investigation using Cramer’s V for correlation between categorical variables or ordinal and categorical variables and Kendall’s Tau B for correlations between ordinal variables. We found that the majority (74%) of the Cramer’s V correlations (*n* = 593) were between 0 and 0.1 (weak association) and the majority (84%) of the Kendall’s Tau B correlations (*n* = 37) were between 0 and 0.3 (weak association) (Akoglu, 2018). Variables were found to be weakly correlated and therefore judged not to be collinear.

We used Stata version 15.1 IC for data analysis. Survey weights were applied by using the svy prefix, and standard errors were calculated using the Taylor series linearization. The overall fit of each model was assessed using the Hosmer and Lemeshow goodness of fit test with a *p*-value of greater than 0.05 considered as a marker of good fit (Hosmer, 2013).

## Results

### Descriptive Analysis

The study sample (*n* = 2080) identified from the CLSA was composed of individuals aged 45 to 85 at baseline who retired between baseline and follow-up one (Table 1). The prevalence of perceived involuntary retirement in the study sample was 28.0% and did not differ between women and men. Most of the sample had post-secondary education, expected their retirement incomes to be adequate, had an employer pension, were in excellent or very good health, and were married. Women and men were similar in most background characteristics. However, women were more likely to have been providing care to others (56 vs. 45%) and men were more likely to have an employer pension (72 vs. 47%). As well, the most common occupation for women was business versus trades for men and the most common industry for women was health care or social assistance versus construction for men.

**Table 1.** Characteristics of Study Sample by Sex.

Domain	Total (*n* = 2080)	CI 95%	Women (*n* = 1115)	CI 95%	Men (*n* = 965)	CI 95%
Involuntarily retired	28.0%	25.1–31.2	28.9%	24.7–33.4	27.1%	23.0–31.6
Sex						
Male	53.9	50.6–57.2	—	—	—	—
Female	46.1	42.8–49.4	—	—	—	—
1. Demographics						
Race						
White	95.3	93.7–96.6	95.6	93.1–97.3	95.0	92.5–96.7
Non-white	4.7	3.4–6.3	4.4	2.7–6.9	5.0	3.3–7.5
Immigrant status						
Immigrant	11.9	10.1–14.1	10.8	8.4–13.6	13.3	10.5–16.7
Non-immigrant	88.1	85.9–89.9	89.2	86.4–91.6	86.7	83.3–89.5
2. Human capital and finances						
Education (highest level)						
Less than high school	15.1	12.1–18.7	15.3	11.1–20.6	14.9	10.9–20.1
High school graduate	14.9	12.7–17.4	16.1	13.0–19.7	13.5	10.5–17.3
Some post-secondary	10.8	9.0–13.0	11.7	9.2–14.8	9.8	7.3–13.0
Post-secondary graduate	59.2	55.7–62.6	57.0	52.2–61.7	61.8	56.7–66.7
Household income						
<$50,000	23.5	20.4–26.8	27.2	22.7–32.4	19.3	15.7–23.4
$50,000 to <$100,000	36.3	33.1–39.6	33.9	29.8–38.4	38.9	34.1–43.8
$100,000 to <$150,000	22.4	19.7–25.3	22.2	18.6–26.3	22.6	18.8–26.9
$150,000 or more	17.9	15.6–20.4	16.6	13.6–20.2	19.2	16.0–23.0
Employer pension benefits	64.3	60.9–67.5	57.2	52.5–61.8	72.1	67.5–76.3
Expected adequacy of retirement income						
Inadequate	10.0	8.1–12.3	10.0	7.4–13.4	9.9	7.3–13.4
Barely adequate	16.4	13.9–19.4	16.7	13.1–21.1	16.2	12.8–20.3
Adequate	73.6	70.3–76.6	73.3	68.5–77.5	73.9	69.3–78.1
3. Work context						
Employment status						
Employed	85.8	83.1–88.1	82.0	77.8–85.5	90.1	86.8–92.6
Unemployed	3.0	2.0–4.5	3.7	2.2–6.3	2.3	1.4–3.7
Unable to work	6.5	5.1–8.2	6.6	4.8–9.0	6.3	4.3–9.1
Non-work activities	4.7	3.2–6.9	7.7	5.2–11.4	1.3	0.5–3.9
Years working at last job						
<1	3.2	2.2–4.6	3.2	2.0–5.2	3.2	1.9–5.5
1 to <3	5.0	3.7–6.6	6.5	4.4–9.3	3.4	2.3–5.1
3 to <5	7.0	5.4–9.0	7.4	5.4–10.1	6.5	4.3–9.9
5+	84.8	82.2–87.1	82.9	79.1–86.1	86.8	83.1–89.8
Occupation						
Management	11.8	9.9–13.9	10.3	8.1–13.1	13.5	10.7–17.0
Business	18.4	16.0–21.1	24.4	20.7–28.4	11.5	8.6–15.1
Sciences	7.0	5.5–8.8	4.2	2.7–6.3	10.3	7.8–13.4
Health	7.2	5.8–8.7	11.4	9.0–14.2	2.3	1.4–4.0
Educ/Law	15.0	13.1–17.1	17.8	15.0–21.1	11.7	9.5–14.4
Art/culture/sports	5.2	3.7–7.3	5.7	3.4–9.3	4.5	2.9–6.9
Sales/service	16.5	14.0–19.2	19.0	15.5–23.0	13.5	10.3–17.5
Trades	12.7	10.6–15.2	2.6	1.6–4.3	24.4	20.3–29.0
Natural resources/agriculture	3.9	2.9–5.3	2.3	1.3–3.9	5.8	4.0–8.3
Manufacturing	2.4	1.2–4.9	2.4	0.7–7.9	2.5	1.3–4.6
Industry						
Agriculture/forestry/fishing	4.7	3.6–6.1	3.8	2.5–5.6	5.8	4.1–8.2
Mining/oil/gas	2.7	2.0–3.7	1.3	0.7–2.6	4.3	3.0–6.1
Utilities	1.8	1.2–2.7	1.5	0.8–2.7	2.1	1.2–3.7
Construction	7.6	5.8–9.8	2.7	1.4–5.1	13.3	10.1–17.4
Manufacturing	6.6	4.7–9.1	3.8	1.6–8.6	9.8	7.1–13.2
Wholesale trade	0.3	0.1–0.7	0.3	0.1–1.0	0.3	0.1–1.1
Retail trade	6.2	4.6–8.2	7.6	5.2–10.8	4.5	2.7–7.4
Transportation	6.8	5.2–8.9	2.7	1.6–4.6	11.6	8.5–15.6
Information/cultural activities	1.9	1.3–2.8	1.9	1.0–3.4	1.9	1.2–3.2
Finance/insurance	4.5	3.5–5.7	4.7	3.4–6.7	4.1	2.9–5.9
Real estate	1.1	0.4–3.0	1.5	0.4–5.6	0.7	0.3–1.6
Prof./scientific/tech services	6.2	4.8–8.1	6.1	4.4–8.5	6.3	4.1–9.6
Management of companies	0.8	0.4–1.5	0.4	0.1–1.3	1.3	0.6–2.6
Administration/waste mgmt.	1.9	1.1–3.3	1.8	0.9–3.6	2.0	0.8–4.9
Education	8.5	7.1–10.1	10.6	8.4–13.2	6.0	4.6–7.9
Health care/social assistance	17.4	15.3–19.9	25.7	22.2–29.6	7.8	5.8–10.4
Arts/entertainment	3.9	2.5–5.9	4.5	2.5–8.0	3.1	1.8–5.4
Accommodations/food service	4.4	3.2–6.0	4.4	3.0–6.6	4.3	2.6–7.1
Other services	2.9	2.0–4.3	4.7	3.0–7.3	0.8	0.4–1.6
Public administration	9.8	8.3–11.6	9.9	7.8–12.4	9.8	7.7–12.4
4. Life-span development						
Self-rated health						
Excellent/very good	56.8	53.4–60.1	58.4	53.8–63.0	54.9	50.0–59.6
Good	30.5	27.5–33.7	27.4	23.4–31.8	34.2	29.8–39.0
Fair/poor	12.7	10.5–15.3	14.2	11.0–18.1	10.9	8.2–14.4
Self-rated mental health						
Excellent/very good	65.2	61.9–68.4	64.2	59.5–68.5	66.4	61.6–70.9
Good	30.4	27.3–33.7	30.8	26.6–35.4	29.9	25.4–34.7
Fair/poor	4.4	3.4–5.7	5.0	3.6–7.0	3.7	2.6–5.4
Number of chronic conditions						
1	25.1	22.4–28.0	22.0	18.6–25.8	28.7	24.7–33.2
2	20.3	17.9–22.9	17.6	14.7–21.0	23.4	19.6–27.6
3–4	27.9	25.0–30.9	26.7	23.0–30.8	29.3	25.0–34.0
5+	26.8	23.7–30.0	33.7	29.2–38.6	18.6	15.2–22.6
Activity limitation (ADLs/IADLs)						
None	92.6	90.5–94.3	90.6	87.4–93.0	95.0	92.0–96.9
Mild, moderate, or severe	7.4	5.7–9.5	9.4	7.0–12.6	5.0	3.1–8.0
Participation limitations						
No desire to participate in more activities	55.7	52.3–58.9	53.1	48.4–57.6	58.7	53.9–63.3
Desire but limited by health	6.8	3–8.7	8.0	5.7–11.2	5.4	3.8–7.6
Desire but limited by other	37.5	34.3–40.8	38.9	34.5–43.6	35.9	31.4–40.7
5. Linked lives						
Marital status						
Married/common law	78.4	75.5–81.1	75.0	70.7–78.9	82.3	78.5–85.6
Single/separated/widowed	21.6	18.9–24.5	25.0	21.1–29.3	17.7	14.4–21.5
Has children	85.0	82.0–87.6	83.9	79.0–87.8	86.4	83.0–89.1
Household size 3 plus	31.3	28.2–34.7	33.5	29.0–38.2	28.9	24.6–33.5
Spouse not retired	72.1	68.8–75.3	72.1	67.4–76.3	72.2	67.2–76.7
Care assistance due to health	50.8	47.4–54.1	55.8	51.2–60.3	44.9	40.1–49.8
Assistance to spouse/partner	6.8	5.2–8.9	5.8	3.8–8.5	8.0	5.6–11.3
Assistance to parent	18.5	16.1–21.1	22.3	18.8–26.2	14.1	11.1–17.6
Assistance to child	2.5	1.7–3.6	3.9	2.6–5.9	0.8	0.4–1.8
Assistance to others	22.8	19.9–25.9	23.6	19.7–28.0	21.8	17.8–26.4
Did not provide any assistance or only provided financial assistance	49.5	46.1–52.8	44.4	39.9–49.1	55.3	50.4–60.0
6. Retirement conditions						
Health/disability/stress	28.5	25.5–31.7	29.7	25.4–34.3	27.1	22.9–31.6
Organizational restructuring	13.5	11.4–16.0	14.2	11.3–17.7	12.8	10.0–16.3
Mandatory retirement policy	1.7	1.1–2.8	1.8	0.9–3.6	1.6	0.8–3.2
Provide care to others	8.8	7.3–10.7	12.1	9.6–15.1	5.0	3.5–7.0
Pension eligible	28.0	25.2–30.9	21.9	18.7–25.5	35.0	30.6–39.7
Financial possibility	53.5	50.1–56.8	50.0	45.4–54.6	57.5	52.8–62.2
Employer incentive	6.8	5.4–8.5	6.4	4.5–9.1	7.2	5.4–9.6
Pursuit of hobbies/other activities	37.5	34.5–40.7	36.7	32.5–41.1	38.5	34.1–43.1
Wanted to stop working	46.0	42.8–49.3	46.4	41.9–51.0	45.6	40.9–50.3
Agreement with spouse	28.5	25.7–31.4	30.0	26.0–34.3	26.7	22.9–30.8
7. Timing, time, and place						
Age at retirement						
<55	24.1	21.2–27.2	29.2	25.0–33.8	18.2	14.7–22.4
55–64	55.0	51.7–58.3	56.1	51.4–60.6	53.8	49.0–58.6
65+	20.9	18.3–23.7	14.7	11.9–18.2	28.0	23.8–32.5
Time since retirement						
<1	16.8	14.5–19.4	17.5	14.4–21.0	16.0	12.8–19.9
1–2	38.4	35.2–41.8	36.4	31.9–41.1	40.8	36.2–45.7
3 years plus	44.8	41.5–48.1	46.2	41.6–50.8	43.1	38.5–47.9
Preparations for retirement						
None	8.5	6.8–10.5	8.8	6.5–11.7	8.2	5.9–11.2
At least some	91.5	89.5–93.2	91.2	88.3–93.5	91.8	88.8–94.1
Region						
Atlantic	7.0	6.0–8.0	6.6	5.4–8.1	7.4	6.0–9.0
Quebec	21.2	18.7–24.1	21.2	17.8–25.2	21.2	17.5–25.4
Ontario	38.0	34.7–41.4	38.0	33.5–42.8	38.0	33.2–43.0
Prairies	16.1	14.1–18.3	15.8	13.1–19.0	16.4	13.7–19.6
British Columbia	17.7	15.3–20.4	18.3	15.0–22.2	17.0	13.7–21.0

Note. Percentages are weighted using CLSA Tracking Cohort inflation weights which account for probability of selection into the sample and survey non-response.

In terms of retirement context, the most common reasons reported for retirement were retiring as it was financially possible, reported by half of the sample, followed by having wanted to stop working (46%), pursuit of hobbies and other activities (38%), health, disability or stress (29%), agreement with spouse (29%), and eligibility for a pension (28%). Around 10%, the sample reported organizational restructuring/job eliminated and care responsibilities as reasons for retirement. Less than 10% of the sample reported retiring due to a mandatory retirement policy and less than 5% reported mandatory retirement as a reason for retirement. Women and men were similar in reasons reported for their retirement, except for eligibility for a pension which was more important among men (35% vs. 22%) and stopping work for care responsibilities which was more commonly reported by women (12% vs. 5%). Over 90% of both women and men had made at least some preparations for retirement. However, men were more likely than women to retire at older ages (65+) (28% vs. 15%).

### Logistic Regression Analyses

The results from the logistic regression analyses are presented in Tables 2 (women) and 3 (men). We produced three sets of models (unadjusted, adjusted across each of seven domains, and adjusted across domains) for women and men separately. In our final model, fourteen factors across four domains were found to be significant. As expected, women and men differed in their predictors on retirement voluntariness with only four of these factors found to be common to both women and men.

**Table 2.** Logistic Regression Models of Involuntary Retirement for Women.

Domain	Model 1: Univariate		Model 2: Multivariable		Model 3: Final Multivariable	
	Unadjusted OR	CI 95%	Adjusted OR per Domain	CI 95%	Fully Adjusted OR	CI 95%
1. Demographics						
Non-white (ref white)	1.38	0.59–3.20	—	—	—	—
Immigrant (ref non-immigrant)	0.75	0.44–1.28	—	—	—	—
2. Human capital and finances						
Education—highest level (ref < high school)						
High school graduate	0.77	0.39–1.52	—	—	—	—
Some post-secondary	0.70	0.34–1.47	—	—	—	—
Post-secondary graduate	0.67	0.37–1.22	—	—	—	—
Household income (ref < $50,000)						
$50,000 to < $100,000	0.65*	0.45–0.95	—	—	—	—
$100,000 to < $150,000	0.62*	0.40–0.95	—	—	—	—
$150,000 or more	0.38*	0.22–0.65	—	—	—	—
No employer pension benefits (ref pension)	1.44*	1.06–1.96	—	—	—	—
Expected adequacy of retirement income (ref adequate)						
Inadequate	5.00*	2.97–8.40	4.99*	2.97–8.40	—	—
Barely adequate	2.90*	1.94–4.33	2.90*	1.94–4.33	—	—
Hosmer–Lemeshow goodness of fit	—	—	Prob > F = 1.000*		—	—
3. Work context						
Employment status (ref employed)						
Unemployed	2.81*	1.21–6.55	2.59*	1.14–5.89	0.68	0.16–2.86
Unable to work	8.33*	4.68–14.83	9.81*	5.37–17.90	2.24	0.99–5.05
Non-work activities	0.36*	0.15–0.82	0.30*	0.11–0.81	0.30*	0.12–0.74
Years at last job (ref 5+)						
<1	1.20	0.47–3.04	—	—	—	—
1 to <3	2.65*	1.38–5.09	—	—	—	—
3 to <5	1.31	0.70–2.46	—	—	—	—
Occupation (ref Educ/Law/Govt)						
Management	1.66	0.96–2.87	1.44	0.75–2.78	1.58	0.69–3.58
Business	1.07	0.68–1.67	1.11	0.63–1.93	1.16	0.60–2.23
Sciences	1.95	0.86–4.43	1.73	0.71–4.21	1.41	0.51–3.89
Health	0.96	0.56–1.66	0.86	0.43–1.72	0.93	0.45–1.94
Art/culture/sports	0.70	0.30–1.64	0.34	0.09–1.25	0.31	0.07–1.25
Sales/service	1.63*	1.01–2.63	1.06	0.54–2.10	0.73	0.38–1.40
Trades	1.11	0.40–3.10	0.59	0.17–2.06	1.04	0.35–3.12
Natural resources/agriculture	0.86	0.27–2.68	1.30	0.25–6.82	1.05	0.33–3.39
Manufacturing	8.92*	2.07–38.4	8.30*	1.31–52.60	10.72*	2.53–45.52
Industry (ref public admin)						
Agriculture/forestry/fishing	1.15	0.48–2.75	1.21	0.34–4.33	—	—
Mining/oil/gas	2.54	0.69–9.30	3.85	1.15–12.90	—	—
Utilities	1.39	0.41–4.76	1.22	0.34–4.39	—	—
Construction	2.02	0.68–6.05	2.64	0.90–7.69	—	—
Manufacturing	3.32*	1.14–9.68	1.72	0.46–6.35	—	—
Wholesale trade	2.01	0.17–23.83	—	—	—	—
Retail trade	2.78*	1.42–5.44	2.52	1.09–5.79	—	—
Transportation	2.61	0.84–8.18	3.71	1.16–11.89	—	—
Information/cultural activities	1.34	0.41–4.34	1.54	0.33–7.20	—	—
Finance/insurance	1.16	0.50–2.72	1.19	0.50–2.82	—	—
Real estate	—	—	—	—	—	—
Professional/scientific/tech services	1.49	0.69–3.22	1.28	0.55–3.00	—	—
Management of companies	—	—	—	—	—	—
Administration/waste mgmt.	1.82	0.50–6.62	1.77	0.43–7.30	—	—
Education	1.11	0.59–2.08	1.38	0.71–2.70	—	—
Health care/social assistance	1.42	0.82–2.44	1.81	0.98–3.34	—	—
Arts/entertainment	0.50	0.14–1.74	0.72	0.14–3.82	—	—
Accommodations/food service	3.24*	1.47–7.13	3.51	1.42–8.70	—	—
Other services	1.09	0.46–2.59	2.17	0.86–5.49	—	—
Hosmer–Lemeshow goodness of fit	—	—	Prob > F = 0.599*		—	—
4. Life-span development						
Self-rated health (ref excellent/very good)						
Good	1.58*	1.11–2.25	1.26	0.85–1.85	1.23	0.77–1.98
Fair/poor	5.49*	3.58–8.40	2.99*	1.80–4.98	2.35*	1.34–4.13
Self-rated mental health (ref excellent/very good)						
Good	1.38	0.99–1.93	—	—	—	—
Fair/poor	3.77*	2.16–6.60	—	—	—	—
Number of chronic conditions	1.20*	1.14–1.27	1.11*	1.04–1.19	—	—
ADL/IADL limitations (ref none)	2.91*	1.86–4.55	—	—	—	—
Participation limitations (ref none)						
Health limited participation	5.04*	2.95–8.61	2.61*	1.47–4.61	—	—
Other limited participation	0.94	0.67–1.30	0.96	0.69–1.34	—	—
Hosmer–Lemeshow goodness of fit			Prob > F = 0.422*			
5. Linked lives						
Marital status (ref married/common law)						
Single/separated/widowed/divorced	1.49*	1.08–2.04	—	—	—	—
Has children (ref no children)	0.61*	0.41–0.91	0.61*	0.41–0.91	—	—
Household size 3 plus (ref <3)	0.69*	0.49–0.98	—	—	—	—
Spouse retired (ref spouse not retired)	0.80	0.53–1.20	—	—	—	—
Care assistance due to health (ref no)	0.91	0.68–1.23	—	—	—	—
Assistance to spouse/partner (ref no)	1.48	0.77–2.85	—	—	—	—
Assistance to parent (ref no)	1.01	0.70–1.48	—	—	—	—
Assistance to child (ref no)	0.92	0.37–2.27	—	—	—	—
Assistance to others (ref no)	0.74	0.49–1.10	—	—	—	—
Hosmer–Lemeshow goodness of fit			Prob > F = 1.000*			
6. Retirement conditions						
Health/disability/stress	4.04*	2.96–5.53	6.33*	4.14–9.70	3.78*	2.31–6.18
Organizational restructuring	6.69*	4.40–10.17	10.59*	6.04–18.57	9.44*	5.39–16.51
Mandatory retirement policy	2.41	0.83–7.03	—	—	—	—
Provide care to others	0.74	0.46–1.22	—	—	—	—
Pension eligible	0.31*	0.19–0.48	—	—	—	—
Financial possibility	0.20*	0.14–0.28	0.53*	0.35–0.79	0.56*	0.37–0.84
Employer incentive	0.94	0.46–1.92	—	—	—	—
Pursuit of hobbies/other activities	0.11*	0.07–0.18	0.25*	0.14–0.46	0.25*	0.14–0.44
Wanted to stop working	0.10*	0.07–0.16	0.19*	0.12–0.31	0.19*	0.12–0.31
Agreement with spouse	0.26*	0.16–0.40	—	—	—	—
Hosmer–Lemeshow goodness of fit	—	—	Prob > F = 0.0024		—	—
7. Timing, time, and place						
Age at retirement (ref 65+)						
<55	1.41	0.88–2.25	—	—	—	—
55–64	1.21	0.81–1.82	—	—	—	—
Time since retirement (ref 3+ years)						
<1 year	0.97	0.64–1.48	—	—	—	—
1–2 years	0.87	0.62–1.21	—	—	—	—
Some preparations for retirement (ref none)	0.70	0.42–1.15	—	—	—	—
Region						
Atlantic	0.88	0.57–1.36	—	—	—	—
Quebec	1.20	0.72–1.98	—	—	—	—
Ontario (ref)		—	—	—		
Prairies	0.91	0.60–1.38	—	—	—	—
British Columbia	1.13	0.67–1.91	—	—	—	—
Hosmer–Lemeshow goodness of fit	—	—	—		Prob > F = 0.534*	

Source. Baseline and follow-up one data from the Canadian Longitudinal Study on Aging Tracking cohort.

*Good fit if *p* > .05 for Hosmer–Lemeshow goodness of fit at the model level.

**Table 3.** Logistic Regression Models of Involuntary Retirement for Men.

Domain	Model 1: Univariate		Model 2: Multivariate		Model 3: Final Multivariate	
	Unadjusted OR	CI 95%	Adjusted OR per Domain	CI 95%	Fully Adjusted OR	CI 95%
1. Demographics						
Non-white (ref white)	1.07	0.46–2.45	—	—	—	—
Immigrant (ref non-immigrant)	1.25	0.75–2.06	—	—	—	—
2. Human capital and finances						
Education—highest level (ref < high school)						
High school graduate	0.46*	0.21–0.98	—	—	—	—
Some post-secondary	0.70	0.31–1.56	—	—	—	—
Post-secondary graduate	0.56	0.30–1.06	—	—	—	—
Household income (ref < $50,000)						
$50,000 to < $100,000	0.45*	0.30–0.70	0.57*	0.36–0.91	—	—
$100,000 to < $150,000	0.31*	0.18–0.54	0.43*	0.24–0.76	—	—
$150,000 or more	0.31*	0.17–0.57	0.46*	0.24–0.87	—	—
No employer pension benefits (ref pension)	1.72*	1.19–2.50	—	—	—	—
Expected adequacy of retirement income (ref adequate)						
Inadequate	6.28*	3.62–10.90	5.15*	2.88–9.21	2.79*	1.43–5.48
Barely adequate	2.26*	1.42–3.60	1.95*	1.21–3.14	1.62	0.91–2.89
Hosmer–Lemeshow goodness of fit	—	—	Prob > F = 0.9996*		—	—
3. Work context						
Employment status (ref employed)						
Unemployed	4.34*	1.89–9.94	4.20*	1.76–10.05	—	—
Unable to work	10.27*	5.37–19.66	10.17*	5.33–19.40	—	—
Non-work activities	0.60	0.12–2.90	0.53	0.11–2.69	—	—
Years at last job (ref 5+)						
<1	2.12	0.91–4.94	—	—	—	—
1 to <3	3.13*	1.53–6.40	—	—	—	—
3 to <5	1.41	0.56–3.54	—	—	—	—
Occupation (ref Educ/Law/Govt)						
Management	1.44	0.70–2.97	1.63	0.77–3.47	—	—
Business	1.20	0.59–2.45	1.31	0.62–2.77	—	—
Sciences	1.72	0.87–3.40	1.64	0.80–3.34	—	—
Health	1.40	0.47–4.17	1.39	0.46–4.22	—	—
Art/culture/sports	1.60	0.63–4.05	1.74	0.68–4.45	—	—
Sales/service	2.30*	1.19–4.46	2.36	1.15–4.83	—	—
Trades	1.60	0.90–2.83	1.40	0.75–2.58	—	—
Natural resources/agriculture	1.15	0.48–2.79	1.36	0.54–3.44	—	—
Manufacturing	4.81*	1.68–13.71	4.42	1.50–13.07	—	—
Industry (ref public admin)						
Agriculture/forestry/fishing	1.25	0.57–2.74	—	—	—	—
Mining/oil/gas	1.72	0.73–4.06	—	—	—	—
Utilities	1.17	0.35–3.92	—	—	—	—
Construction	1.26	0.55–2.89	—	—	—	—
Manufacturing	1.81	0.88–3.75	—	—	—	—
Wholesale trade	0.67	0.06–7.04	—	—	—	—
Retail trade	1.70	0.70–4.16	—	—	—	—
Transportation	0.91	0.39–2.11	—	—	—	—
Information/cultural activities	1.42	0.47–4.26	—	—	—	—
Finance/insurance	1.51	0.58–3.95	—	—	—	—
Real estate	0.23	0.03–1.96	—	—	—	—
Professional/scientific/tech services	1.33	0.57–3.11	—	—	—	—
Management of companies	2.38	0.56–10.04	—	—	—	—
Administration/waste mgmt.	0.84	0.16–4.50	—	—	—	—
Education	0.67	0.32–1.41	—	—	—	—
Health care/social assistance	0.87	0.40–1.87	—	—	—	—
Arts/entertainment	1.58	0.50–5.00	—	—	—	—
Accommodations/food service	1.16	0.43–3.12	—	—	—	—
Other services	0.88	0.22–3.57	—	—	—	—
Hosmer–Lemeshow goodness of fit	—	—	Prob > F = 1.000*		—	—
4. Life-span development						
Self-rated health (ref excellent/very good)						
Good	1.20	0.81–1.78	1.12	0.74–1.69	—	—
Fair/poor	3.20*	1.94–5.28	2.40*	1.42–4.06	—	—
Self-rated mental health (ref excellent/very good)						
Good	1.37	0.93–2.02	—	—	—	—
Fair/poor	2.77*	1.44–5.32	—	—	—	—
Number of chronic conditions	1.09*	101–1.18	—	—	—	—
ADL/IADL limitations (ref none)	3.85*	1.99–7.42	2.42*	1.23–4.77	—	—
Participation limitations (ref none)						
Health limited participation	5.22*	2.57–10.57	3.48*	1.69–7.19	2.75*	1.15–6.53
Other limited participation	1.62*	1.11–2.35	1.63*	1.11–2.40	1.22	0.76–1.96
Hosmer–Lemeshow goodness of fit	—	—	Prob > F = 0.9976*		*--*	
5. Linked lives						
Marital status (ref married/common law)						
Single/separated/widowed/divorced	2.10*	1.43–3.10	2.18*	1.47–3.24	—	—
Has children (ref no children)	0.92	0.56–1.50	—	—	—	—
Household size 3 plus (ref <3)	0.87	0.58–1.31	—	—	—	—
Spouse retired (ref spouse not retired)	0.89	0.56–1.41	—	—	—	—
Care assistance due to health (ref no)	1.32	0.93–1.88	—	—	—	—
Assistance to spouse/partner (ref no)	2.01*	1.13–3.59	2.24*	1.24–4.05	—	—
Assistance to parent (ref no)	1.45	0.87–2.41	1.51	0.90–2.55	—	—
Assistance to child (ref no)	1.72	0.41–7.24	1.55	0.39–6.18	—	—
Assistance to others (ref no)	0.97	0.62–1.52	0.96	0.61–1.51	—	—
Hosmer–Lemeshow goodness of fit	—	—	Prob > F = 0.999*		—	—
6. Retirement conditions						
Health/disability/stress	3.30*	2.28–4.77	4.15*	2.57–6.69	3.00*	1.82–4.96
Organizational restructuring	6.12*	3.79–9.87	10.28*	5.39–19.60	11.31*	5.58–22.92
Mandatory retirement policy	6.85*	2.23–21.02	11.92*	2.28–62.4	15.03*	2.23–101.32
Provide care to others	1.17	0.58–2.35	—	—	—	—
Pension eligible	0.25*	0.16–0.38	0.44*	0.25–0.77	0.37*	0.21–0.67
Financially possible	0.22*	0.15–0.33	0.36*	0.21–0.63	0.39*	0.22–0.68
Employer incentive	1.79*	1.03–3.12	—	—	—	—
Pursuit of hobbies/other activities	0.24*	0.15–0.38	0.55	0.30–1.00	0.57	0.30–1.08
Wanted to stop working	0.17*	0.10–0.28	0.36*	0.21–0.63	0.45*	0.25–0.80
Agreement with spouse	0.35*	0.20–0.61			—	—
Hosmer–Lemeshow goodness of fit	—	—	Prob > F = 0.088*		—	—
7. Timing, time, and place						
Age at retirement (ref 65+)						
<55	4.10*	2.42–6.94	4.01*	2.34–6.89	2.49*	1.25–4.96
55–64	1.82*	1.17–2.85	2.15*	1.35–3.42	2.64*	1.46–4.75
Time since retirement (ref 3+ years)						
<1 year	0.63	0.35–1.14	—	—	—	—
1–2 years	0.63*	0.43–0.91	—	—	—	—
Preparations for retirement (ref none)						
At least some	0.21*	0.12–0.36	0.22*	0.12–0.39	0.34*	0.18–0.64
Region						
Atlantic	0.87	0.55–1.37	—	—	—	—
Quebec	1.00	0.59–1.69	—	—	—	—
Ontario (ref)			—	—		
Prairies	0.96	0.57–1.61	—	—	—	—
British Columbia	0.82	0.46–1.46	—	—	—	—
Hosmer–Lemeshow goodness of fit	—	—	Prob > F = 1.000*		Prob > F = 0.417*	

Source. Baseline and follow-up one data from the Canadian Longitudinal Study on Aging Tracking cohort.

*Good fit if *p* > .05 for Hosmer–Lemeshow goodness of fit at the model level.

As expected, finances and education predicted retirement voluntariness. In unadjusted models (Model 1), women and men without an employer pension and with expectations of inadequate and barely adequate income prior to retirement had greater odds of involuntary retirement. On the other hand, those with higher incomes and men with high school education compared to less than high school education had greater odds of voluntary retirement. Education and employer pension were no longer significant after adjusting across human capital and finances factors and, therefore, indirectly predicted retirement voluntariness (Model 2). However, women and men who expected their incomes to be barely adequate or inadequate had two to five times greater odds of involuntary retirement and income remained significant among men. In our final model, only expectations of inadequate income for retirement among men remained significant, at three times greater odds of involuntary retirement.

Several work context factors predicted retirement voluntariness in unadjusted models, but only one remained in our final model. As expected, being unable to work (women and men) and job loss prior to retiring (women and men) were associated with greater odds of involuntary retirement. In addition, having worked at their last job for a shorter period (women), occupation (sales/service and manufacturing among women and men), and industry (manufacturing, retail trade, or accommodations/food service among women) were associated with greater odds of involuntary retirement. However, having been involved in non-work activities such as volunteering was associated with greater odds of voluntary retirement among women. In models adjusted across these factors, occupation and industry were no longer significant. Only having worked in a manufacturing occupation among women remained significant in our final model, which was associated with 11 times greater odds of involuntary retirement compared to occupations in education/law/government.

Also, as expected, poorer health and disability predicted retirement voluntariness. Poorer self-rated health and mental health (women and men), greater number of chronic conditions (women and men), ADL/IADL limitations (women and men), and having participation limitations (women and men) were associated with greater odds of involuntary retirement. However, self-rated mental health, chronic conditions, and ADL/IADL limitations were no longer significant after adjusting across life-span development factors. In our final model, only poorer self-rated health among women and health-related participation limitations among men remained significant, both at two times greater odds compared to those with excellent or very good health and those without participation limitations.

Several linked lives factors were significant in unadjusted models, but none remained so in our final model. Men who had care responsibilities for a spouse or partner had greater odds of involuntary retirement. However, women who had children and lived in a larger household had greater odds of voluntary retirement. Unexpectedly, spousal retirement status was not a predictor. After adjusting across these factors, only having care responsibilities among men remained significant. In our final model, this factor was no longer significant.

Retirement conditions proved to be important in directly predicting retirement voluntariness, with many factors remaining significant in our final model. The greatest odds of involuntary were among women and men who reported organizational restructuring/job elimination (9 and 11 times greater odds) and among men who reported retiring due to a mandatory retirement policy (15 times). Among both women and men, retiring due to health, disability, or stress was associated with three times greater odds of involuntary retirement. On the other hand, greater odds of voluntary retirement were found among women and men who reported that retiring was financially possible (two and three rimes), women and men who reported having wanted to stop working (two and five times), men who reported retiring as they were eligible for a pension (three times), and women who reported retiring to pursue hobbies or other activities (four times). Further, we expected those who retired due to caregiving responsibilities and those who lived in Atlantic Canada to have greater odds of involuntary retirement, but neither was significant.

Timing, time, and place factors were significant for men but not women. As expected though, men who were younger at retirement and retired more recently had greater odds of involuntary retirement while those who had prepared for retirement had greater odds of voluntary retirement. Time since retirement was no longer significant after adjusting for preparations for retirement and age at retirement. In our final model, younger age at retirement and preparations for retirement remained significant. Men who retired younger than age 65 had two to three times greater odds of involuntary retirement while those who prepared for retirement had three times greater odds of voluntary retirement.

In our final model, four of fourteen factors were common to both women and men: retiring because of organizational restructuring; because of health, disability, or stress; having wanted to stop working; and retirement being financially possible. The extent of sex-specific predictors suggests that gender norms influence the perception of involuntary retirement. For women, reporting fair or poor health prior to retirement (life-span development) increased their odds of involuntary retirement while being involved in non-work activities such as caring for others and volunteering prior to retirement (work context) and retiring to pursue hobbies (retirement conditions) lowered the odds of involuntary retirement. For men, reporting having inadequate income for retirement prior to retirement (human capital and finances), retiring because of a mandatory retirement policy (retirement conditions), and retiring prior to age 65 (timing time and place) increased the odds of involuntary retirement while retiring due to being eligible for a pension or having prepared for retirement (retirement conditions) lowered the odds of involuntary retirement.

## Discussion

We sought to develop a more comprehensive theoretical model of retirement voluntariness than previous models and to test this model among women and men in Canada using nationally representative longitudinal data of predominately (84%) baby boomers born between 1946 and 1964 (Dimock, 2022). Many early baby-boomer retirees in the United States, that is, those who retired during the Great Recession of 2007 to 2009, have been found to leave their career jobs involuntarily, with layoffs being a key factor (Cahill et al., 2015). However, our study sample retired after the recession (between 2011 and 2018), a time during which the economy was consistently improving, and labor market demand was strengthening in Canada. Future generations of retirees could face a different retirement context. However, the predictors of retirement voluntariness, while varying in their magnitude of impact across generations, are likely to remain the same in the future.

Our theoretical model included seven domains (five background and two retirement context domains) aligned with the principles of the life course perspective. Built following a review of the literature and previous models, that theoretical model included 53 factors, of which 37 were examined in this study. More than one-quarter of recent retirees perceived their retirement as involuntary. Further, among our sample of recent retirees (involuntarily and voluntarily retired), the most common reasons for retirement were financial possibility followed by wanting to stop working, pursuit of hobbies or other activities, disability, health or stress, agreement with spouse, and pension eligibility. Many factors across six of seven domains either directly or indirectly explained perceived retirement voluntariness.

### Policy and Practice Implications

There were several policy implications related to our findings. Job loss factors were significant predictors for retirement voluntariness, as was the case in previous studies (Ebbinghaus & Radl, 2015; Humble et al., 2012; Szinovacz & Davey, 2005; van Solinge, 2007). Over one in ten of our study sample experienced job loss (i.e., unemployment prior to retirement, or retired due to organizational restructuring/job elimination, an employer incentive, or a mandatory retirement policy) which was associated with up to fifteen times greater odds of involuntary retirement, suggesting an important area for policy and practice intervention. Organizations may use restructuring or incentives to shed older workers, thus increasing their feelings of having been forced out. Further, while as of 2009 all provinces had enacted legislation to eliminate mandatory retirement age, there are still some occupational exceptions, such as military personnel, police, and pilots (Berger, 2012, 2021). These situations may stem from ageism, often coupled with ableist attitudes, which can impact the retention of older workers (Berger, 2012, 2021; WHO, 2021). Therefore, organizations and governments should consider ways of combatting these attitudes. In addition, older workers experience greater difficulty finding new employment once unemployed as compared to younger workers (Berger, 2012, 2021). As bridge employment has been found to mitigate poor outcomes associated with involuntary retirement (Dingemans & Henkens, 2014), governments and employers should consider providing employment supports to older workers facing job loss.

Consistent with previous studies, we found that disability/health/stress, as well as caregiving responsivities prior to retirement, predicted perceived retirement voluntariness. Given that almost one-third of our study sample cited disability/health/stress as a reason for retirement resulting in three times greater odds of involuntary retirement and over half the sample provided care prior to retirement, which doubled the odds of involuntary retirement among men, these circumstances warrant further policy attention. Governments need to consider that faced with these barriers to continued work, many older workers may not be responsive to policies aimed at delaying retirement, such as increasing the age of eligibility for national pension benefits. These workers may face financial hardship and turn to unemployment or disability benefit programs, possibly offsetting any savings from these policies (Szinovacz & Davey, 2005). Therefore, it is recommended that, instead, policy makers and employers address barriers to continued work. Doing so could include flexibility with respect to working hours, work arrangements, and supporting pension systems which could help those experiencing work disability (Rouzet et al., 2019) as well as those with caregiving responsibilities (Humble et al., 2012; Rouzet et al., 2019). Moreover, there is considerable evidence of the effectiveness of case management, work accommodations, and multidisciplinary health care for the prevention of work disability aimed at older workers specifically (Steenstra et al., 2017) as well as the general population of workers (Cancelliere et al., 2016; de Boer et al., 2015; Kamper et al., 2015; Nieuwenhuijsen et al., 2014). Better yet, health promotion measures such as those that address tobacco use, weight management, and physical activity have been found to be effective in improving mental and physical health (Cooklin et al., 2017) and therefore have potential to protect older workers from experiencing work disability and subsequent involuntary retirement.

We found that finances did indeed predict retirement voluntariness. Prior to retirement, expectations of barely or inadequate retirement income, reported by about one in ten of our study sample, predicted three to five times greater odds of involuntary retirement. On the other hand, after having retired, reporting retiring due to it being financially possible and because of being eligible for a pension was associated with greater odds of voluntary retirement among both women and men. With about one-third of our sample being ineligible for an employer pension, policy makers and practitioners should consider providing workers with financial education; those who are financially literate have been found to have greater postretirement savings (Lusardi & Mitchell, 2007). Further, these supports should be targeted to those with lower education and income, for whom financial education has been found to be most effective (Lusardi, 2004). Moreover, governments and employers can encourage retirement savings through structural changes (e.g., enhance tax benefits, raise retirement plan contribution limits, and allow enrollment by default) or through persuasive communications (Wiener & Doescher, 2008).

Another avenue that may protect workers from retiring involuntarily is the pursuit of hobbies or other activities in retirement. More than one-third of our study sample reported pursuit of hobbies or other activities as a reason for retirement, which was found to be associated with four times greater odds of voluntary retirement among women. As the importance of having a leisure activity in retirement, particularly social activities on well-being, has been well documented (Adams et al., 2011; Litwin & Shiovitz-Ezra, 2006), doing so should be encouraged among retirees.

This study revealed that women and men have little in common when it comes to the factors that explain their perception of involuntary retirement. We found only four of fourteen direct predictors were common to both women and men. Some sex-specific predictors suggest that gender norms influence the perception of retirement voluntariness. For example, among women but not men, those who retired to pursue hobbies or other activities had greater odds of voluntary retirement. On the other hand, among men but not women, those who retired before the age of 65 or expected inadequate finances for retirement had greater odds of involuntary retirement. These findings might be explained by work and breadwinning playing a more central role in the identities of men than for women and the traditional role of women as caregivers (Berger, 2021; Humble et al., 2012). As such, this result suggests the need for support that is sensitive to these differences. This could include ensuring that pre-retirement planning courses include the importance of having a purpose in retirement, which appears to be more important among women, in addition to financial planning which appears to be more important among men. Moreover, providing bridge employment supports to men under the age of 65 could help to lower their odds of involuntary retirement.

### Further Research

Further research is needed in several areas on this important topic, particularly regarding the design of surveys. The first involves how voluntariness is operationalized in surveys. The CLSA measure of voluntariness is dichotomous rather than as a matter of degree of voluntariness as it was originally conceived (Beehr, 1986; MacLean et al., 2022). In contrast, retirement surveys in the United States and Australia use a measure with three levels of voluntariness: wanted to retire, part wanted, part forced or forced into retirement. Individuals who feel that their retirement was partly forced could be amenable to remaining in the workforce if barriers to continued work were addressed. Further research is needed on how individuals view the extent of voluntariness in retirement and on barriers to continued labor market participation.

A few variables relevant to the study of retirement were not available in the CLSA. Such variables include workplace support or lack of support for remaining at work, which could potentially indicate ageism (Berger, 2021), work stress, which has been found to predict involuntary retirement among women (Szinovacz & Davey, 2005), and lack of work control, which has been linked to work stress and is an important factor leading to disability retirement (Knardahl et al., 2017). This may shed some light on our finding that among women having worked in a manufacturing occupation was directly associated with greater odds of involuntary retirement, as we could not control for these factors. Further research is needed on the feasibility of adding these variables to the CLSA.

The CLSA includes an item that inquires as to the retiree’s reasons for retirement, we have previously discussed policy implications related to a few of these reasons. However, it is difficult to provide advice regarding the response category “wanted to stop working.” Further, as noted by Hewko et al. (2018), this phrase appears to be a “catch-all” response. Therefore, this response is worthy of further study.

### Strengths and Limitations

A major strength of this study is the development and use of a theoretical model of the predictors of retirement voluntariness, rather than involuntary retirement as previous models have done. This focus on the outcome of voluntariness resulted in a more comprehensive model by allowing for the inclusion of factors generally associated with voluntary retirement, such as pursuing hobbies, missing from previous models. We also accounted for the strengths and weaknesses of previous models and used a recent systematic review on predictors of involuntary retirement (MacLean et al., 2022) which resulted in increasing the number of predictors from 20 in the most comprehensive model (Szinovacz & Davey, 2005) to 53. Using the life course perspective principles to group these factors into domains provided a framework for understanding this important life course process. Our model was then tested using a large sample of recent retirees from longitudinal nationally representative data. Further, our analysis of this data was disaggregated, revealing considerable difference between women and men.

Some limitations should be kept in mind when considering the findings of this study. Our theoretical model was based on a small number of studies of predictors of involuntary retirement. Further, some groups were excluded from the CLSA at baseline, such as those with cognitive impairment. While these exclusions represent a small proportion of the Canadian population, given that disability is a predictor of involuntary retirement, those with cognitive impairment may be more likely to retire involuntarily. There is also the risk that some of our models were underpowered, given the relatively low number of cases per indicator. However, most models were well within the widely accepted rule of thumb of 10 cases per indicator variable (Nunnally, 1967). That said, some results should be viewed with caution as cell counts were small for some factors (i.e., having retired due to a mandatory retirement policy among men and being involved in a non-work activity among women) and may explain the lack of significant findings for race and immigration status. Finally, while we did examine sex and binary gender (women and men), we are not able to speak to predictors for those with diverse gender identities.

## Conclusion

More than one-quarter (28%) of older workers perceived their retirement to be involuntary. Among 37 possible predictors across seven domains, 14 factors directly predicted retirement voluntariness and four were common to both women and men. Women and men who reported retiring because of organizational restructuring/job elimination or disability, health, or stress were more likely to perceive their retirement as involuntary, while those who reported retiring as it was financially possible or because they wanted to stop working were more likely to retire voluntarily. Additional predictors among women were occupation (manufacturing), health (self-perceived fair or poor health), employment status prior to retirement (non-work activities such as caring for family and volunteering), and the pursuit of hobbies or other activities while additional predictors among men included expectations of income in retirement, disability status (health-related participation limitations) prior to retirement, mandatory retirement, pension eligibility, age at retirement (<age 65), and preparations for retirement. These findings suggest the need for employment support for those facing job loss, health promotion, work disability prevention interventions (accommodations, case management, and multidisciplinary health care), financial education, and support that is sensitive to the differences between women and men to prevent involuntary retirement.
